# Carboxylated Graphene: An Innovative Approach to Enhanced IgA-SARS-CoV-2 Electrochemical Biosensing

**DOI:** 10.3390/bios15010034

**Published:** 2025-01-09

**Authors:** Luciana de Souza Freire, Ariamna María Dip Gandarilla, Yonny Romaguera Barcelay, Camila Macena Ruzo, Barbara Batista Salgado, Ana P. M. Tavares, Francisco Xavier Nobre, Julio Nino de Souza Neto, Spartaco Astolfi-Filho, Ștefan Țălu, Pritesh Lalwani, Niranjan Patra, Walter Ricardo Brito

**Affiliations:** 1 LABEL–Laboratório de Bioeletrônica e Eletroanalítica, Central Analítica Multidisciplinar, Universidade Federal do Amazonas, Manaus 69067-005, Amazonas, Brazil; lsfreire@ufam.edu.br (L.d.S.F.); ariamna@ufam.edu.br (A.M.D.G.); yonny.barcelay@uc.pt (Y.R.B.); camilarz@ufam.edu.br (C.M.R.); 2 Instituto Leônidas e Maria Deane (ILMD), Fiocruz Amazônia, Manaus 69029-520, Amazonas, Brazil; barbara.salgado@fiocruz.br (B.B.S.); pritesh.lalwani@fiocruz.br (P.L.); 3 CENTI, Centro de Nanotecnologia e Materiais Técnicos, Funcionais e Inteligentes, R. Fernando Mesquita 2785, 4760-034 Vila Nova de Famalicão, Portugal; ana.p.tavares90@gmail.com; 4 Group of Energy Resources and Nanomaterials (GREEN Group), Department of Chemistry, Environment, and Food (DQA), Federal Institute of Education, Science and Technology of Amazonas, Campus Manaus Centro, Manaus 69020-120, Amazonas, Brazil; francisco.nobre@ifam.edu.br; 5 PPGBIOTEC—Programa de Pós-Graduação em Biotecnologia, Universidade Federal do Amazonas, Manaus 69067-005, Amazonas, Brazil; ninobio@ufam.edu.br (J.N.d.S.N.); spartaco.biotec@gmail.com (S.A.-F.); 6 The Directorate of Research, Development and Innovation Management (DMCDI), The Technical University of Cluj-Napoca, 400020 Cluj-Napoca, Romania; 7 Department of Chemistry, Koneru Lakshmaiah Education Foundation, Greenfield, Vaddeswaram 522502, Andhra Pradesh, India; patraji@gmail.com

**Keywords:** carboxylated graphene, screen-printed carbon electrodes, electrochemical biosensors, IgA-SARS-CoV-2, COVID-19

## Abstract

Biosensors harness biological materials as receptors linked to transducers, enabling the capture and transformation of primary biorecognition signals into measurable outputs. This study presents a novel carboxylation method for synthesizing carboxylated graphene (CG) under acidic conditions, enhancing biosensing capabilities. The characterization of the CG was performed using scanning electron microscopy (SEM), energy-dispersive X-ray spectroscopy (EDS), Raman spectroscopy, thermogravimetric analysis (TGA), and X-ray diffraction (XRD). We modified screen-printed carbon electrodes (SPCEs) with CG to immobilize the SARS-CoV-2 N-protein, facilitating targeted detection of IgA antibodies (IgA-SARS-CoV-2). The analytical performance was assessed via electrochemical techniques such as cyclic voltammetry and electrochemical impedance spectroscopy, confirming CG synthesis effectiveness and biosensor functionality. The developed biosensor efficiently detects IgA-SARS-CoV-2 across a dilution range of 1:1000 to 1:200 *v*/*v* in a phosphate-buffered saline (PBS) solution, with a limit of detection calculated at 1:1601 *v*/*v*. This device shows considerable potential because of its fast response time, miniaturized design facilitated by SPCEs, reduced sample volume requirements, high sensitivity and specificity, low detection limits, and signal enhancement achieved through nanomaterial integration.

## 1. Introduction

In recent years, electrochemical biosensors have emerged as a viable alternative to conventional methods, offering numerous advantages such as low cost, portability, low detection limits, stability, and the ability to incorporate nanomaterials into their structure to enhance electrochemical signals. This enhancement increases both sensitivity and specificity. Consequently, the development of electrochemical biosensors has accelerated significantly, and their applications are expanding across various sectors, including industrial, environmental, and biomedical fields [1].

One of the fundamental components of an electrochemical biosensor is undoubtedly the electrode, which supports the system, focusing on applying materials that enhance the mechanical, electrical, and thermal properties. Various electrode types are used as supports in electrochemical biosensor production, including carbon paste, glassy carbon, gold, and screen-printed electrodes. Among these, screen-printed electrodes are particularly notable for their advantages in handling microvolumes of samples and their adaptability to portable devices [1,2,3,4].

Composites have been extensively utilized in producing electrochemical sensors because of their ability to enhance chemical, physical, and magnetic properties. This enhancement increases efficiency and sensitivity in detection systems [5,6]. Among these composites, carboxylated graphene is a prominent type of chemically modified graphene. It consists of graphite structures composed of a layer of graphene functionalized with hydroxyls, epoxides, carbonyls, and carboxylic groups. The use of carboxylated graphene for electrode functionalization is based on several advantages, including excellent optical properties, a high surface-to-volume ratio, outstanding charge mobility, and superior electrical and thermal properties compared with other carbon allotropes. The benefits of carboxylated graphene in developing biosensors include its conductivity, large surface area, and carboxylic groups in its structure, which can act as anchoring points for biomolecules [7,8].

Coronaviruses are a group of positive-sense single-stranded RNA viruses that can infect humans and animals, leading to various diseases that affect the respiratory, gastrointestinal, neurological, and hepatic systems. The symptoms of these infections can range from mild to severe, with some resulting in acute or chronic complications. In 2019, pneumonia cases of unknown origin were reported in the Chinese province of Wuhan. These cases were subsequently linked to the emergence of a new coronavirus, SARS-CoV-2, which belongs to the beta-coronavirus group [9]. As the virus spreads, the demand for rapid and accurate diagnostic tests has become increasingly evident [10]. For the detection of the virus that causes COVID-19, RT-PCR (Reverse Transcription Polymerase Chain Reaction) is considered the gold standard. This test is typically performed in the early stages of the disease when the viral load in the respiratory tract is high; however, it requires specialized laboratories and trained personnel and can be costly. Alternative methods are emerging as viable solutions to overcome the limitations and high costs of RT-PCR tests. Point-of-care testing is a promising approach because it provides rapid results and can be cheaper than other traditional techniques (such as PCR or enzyme-linked immunosorbent assay (ELISA)) [11].

This paper presents the synthesis of carboxylated graphene using a straightforward method, examining its morphological, spectroscopic, and electrochemical characteristics. The synthesized product was also fabricated as an electrochemical biosensor for IgA-SARS-CoV-2 detection.

## 2. Materials and Methods

### 2.1. Reagents

The following reagents were used: nitric acid (H_2_NO_3_), sulfuric acid (H_2_SO_4_), monosodium dihydrogen phosphate (NaH_2_PO_4_), disodium hydrogen phosphate (Na_2_HPO_4_), potassium chloride, *N*-hydroxysuccinimide ester (NHS), 1-ethyl-3-[3-(dimethylamino)propyl]-carbodiimide (EDC), and bovine serum albumin (BSA), all sourced from Sigma-Aldrich and Merck (Rahway, NJ, USA). Graphene nanoplatelets were obtained from XG Sciences (East Lansing, MI, USA), and the 1-Step™ Ultra TMB-ELISA substrate was procured from Thermo Fisher™ Scientific (Waltham, MA, USA). The N-protein was synthesized and purified at the Federal University of Amazonas. Additionally, a solution of 5 mmol L⁻¹ K₃Fe(CN)_6_/K_4_Fe(CN)_6_ prepared in 0.1 mol L⁻¹ KCl was utilized as a redox probe for electrochemical measurements. All electrochemical experiments were performed at room temperature (22 ± 0.5 °C) without stirring. The Federal University of Amazonas provided human blood serum samples, and the study received approval from The Research Ethics Committee of the Federal University of Amazonas (CAAE: 34906920.4.0000.5020), adhering to Brazilian law and the Declaration of Helsinki.

### 2.2. Apparatus and Procedures

Carboxylated graphene (CG) was characterized using several analytical techniques. Thermogravimetric Analysis (TGA) was performed on a Shimadzu TGA-50H instrument in a nitrogen atmosphere, with a flow rate of 50 mL/min and a heating rate of 10 °C/min.

Scanning Electron Microscopy (SEM) and Energy Dispersive X-ray Spectroscopy (EDS) analyses were conducted using a Tescan Vega 3 (Brno, Czech Republic) instrument equipped with an INCA energy system, operating at an accelerating voltage of 15 kV.

X-ray Diffraction (XRD) analyses were performed using a Miniflex II Shimadzu (Nakagyo-ku, Kyoto, Japan) instrument with a Cu Kα radiation source, scanning from 2° to 90° at a speed of 2°/min. Raman Spectroscopy was conducted with a Horiba Scientific MacroRam spectrometer (Minami-ku, Kyoto, Japan), utilizing a 785 nm laser.

Electrochemical measurements were carried out using an Metrohm Autolab PGSTAT204 potentiostat/galvanostat (Utrecht, The Netherlands) equipped with the FRA32M EIS module and NOVA software version 2.1.4. Commercial disposable screen-printed carbon electrodes (SPCEs, refer to product 110) from Metrohm Dropsens (Utrecht, The Netherlands) served as the conductive substrate, with each SPCE comprising a carbon working electrode, a carbon counter electrode, and a pseudo-reference silver electrode. The electrochemical performance was evaluated using cyclic voltammetry (CV) and electrochemical impedance spectroscopy (EIS) with [Fe(CN)_6_]^3^⁻/^4^⁻ as the redox probe. CV was conducted within a potential range of −0.6 to 0.6 V at a scan rate of 50 mV/s, while EIS measurements were performed across a frequency range of 10 kHz to 0.1 Hz at a potential of 0.15 V and an amplitude of 5 mV.

### 2.3. Synthesis of Carboxylated Graphene

**Scheme 1:**
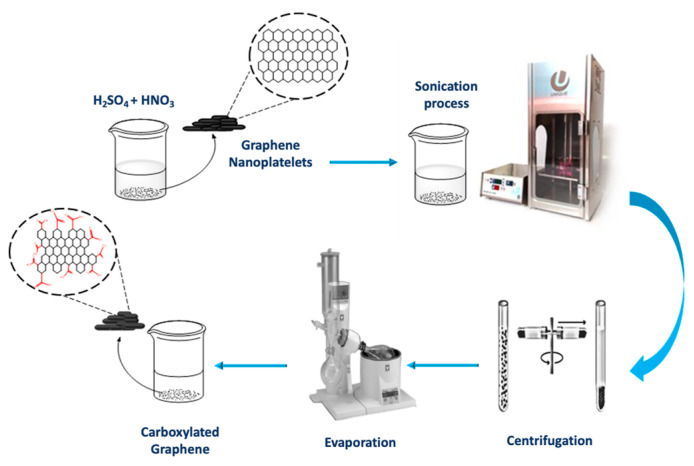
Representation of the method of synthesis of carboxylated graphene.

Carboxylated graphene was synthesized via a carboxylation method in an acidic medium (see Scheme 1). Initially, 7 mg of graphene was dispersed in 30 mL of concentrated H_2_SO_4_, while 3 mg was added to 10 mL of concentrated HNO_3_. The two solutions were mixed and sonicated for 1 h. The resulting modified graphene was washed with Milli-Q water five times, with each step involving centrifugation at 22,000 rpm for 1 h until a neutral pH of 7 [12,13] was achieved. The final product was dried using a rotary evaporation system.

Before modification, the carbon surface was electrochemically activated using 0.5 mol L⁻¹ H_2_SO_4_ via the CV technique. This involved applying various potentials between −1.5 and 1 V at a scan rate of 100 mV s^−1^ for five cycles [14,15]. To prepare the CG/SPCE, the surface of the working electrode (WE) was modified with layers of CG by depositing 5 µL of a CG solution (2 mg mL^−1^) onto the WE surface via drop-casting, followed by drying at 60 °C.

### 2.4. Fabrication of the Biosensor Using CG

The biosensor for detecting IgA of SARS-CoV-2 was constructed following the methodology outlined by Freire et al. [16]. The SPCEs were modified with three layers of CG. Next, the N-protein of SARS-CoV-2 was immobilized onto the modified electrodes using a coupling agent composed of EDC and NHS. This approach detected SARS-CoV-2 antibodies (IgA type) in human serum samples.

The detection process mirrored an ELISA protocol [17]. Specifically, 7 µL of a 1:400 *v*/*v* dilution of human serum containing IgA-SARS-CoV-2 was deposited onto the WE and incubated in a humid chamber for 30 min. After incubation, the immunosensor was treated with horseradish peroxidase-labeled anti-IgA (1:300 *v*/*v*) for 20 min. Following each modification step, the electrode was gently washed with phosphate-buffered saline (PBS) and Milli-Q water. 3,3′,5,5′-tetramethylbenzidine (TMB) was utilized for the enzymatic reaction. 100 µL of TMB was applied to all three electrodes (working, reference, and auxiliary) and incubated in the dark for 2 min. The chronoamperometric (CA) technique monitored the electrochemical response at −0.19 V for 50 s.

## 3. Results

### 3.1. Thermogravimetric Analysis (TGA)

**Figure 1:**
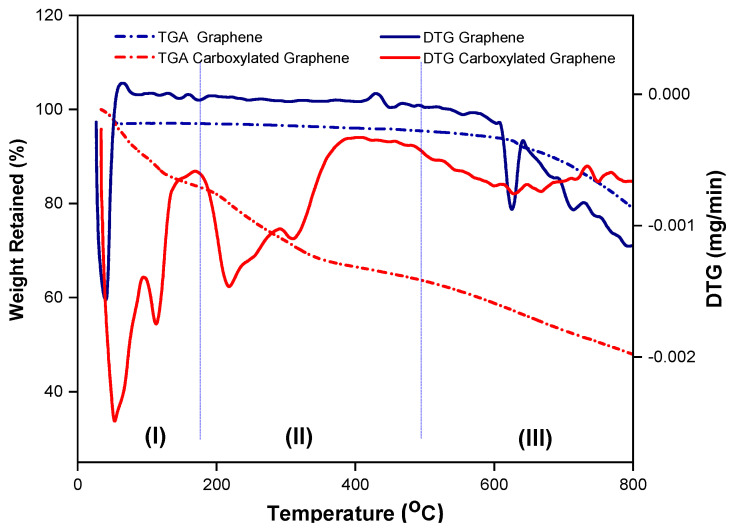
TG and DTG curves of decomposition of graphene and carboxylated graphene.

The comparative Thermogravimetric Analysis (TGA) of graphene and carboxylated graphene is illustrated in Figure 1. The decomposition process is observed in three main steps: (I) evaporation of solvent and water, (II) decomposition of oxygenated functional groups, and (III) formation of carbon monoxide (CO). In the first step, occurring between 0 °C and 170 °C, the mass loss is approximately 3% for graphene and 14% for carboxylated graphene. These losses are attributable to the evaporation of solvent and constitutional water and the adsorption of gases on the material’s surface during the experiment. The second stage, recorded between 170 °C and 610 °C, demonstrates a more significant mass loss of 48% for carboxylated graphene. In this temperature range, organic materials are decomposed into carbonates. The carbonization of organic cations is considered the primary competitive reaction during the decomposition of organic matter due to the oxidation of organic compounds in the air. In the final step, a small weight loss is noted in the temperature range of 610 °C to 800 °C, with approximately 1% for graphene and 14% for carboxylated graphene, which may be associated with the decomposition of CO species [18].

### 3.2. X-Ray Diffraction Analysis (DRX)

**Figure 2:**
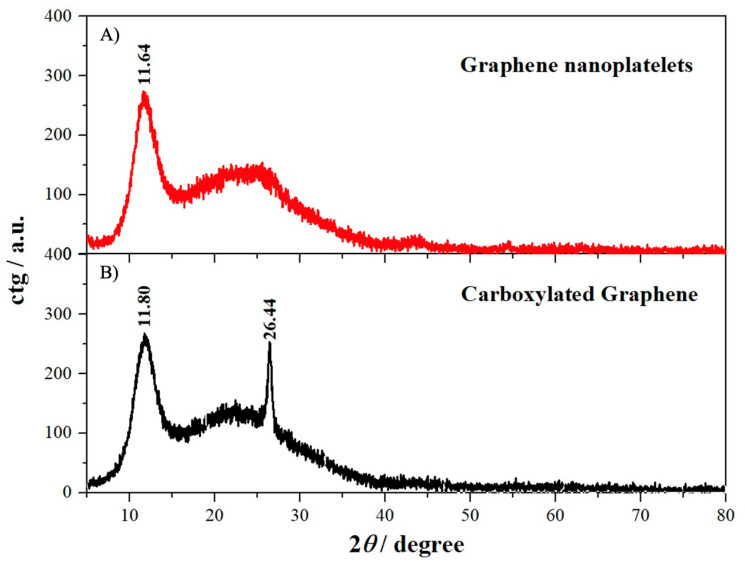
X-ray diffraction pattern of graphene nanoplateles (**A**) and carboxylated graphene (**B**).

Figure 2A,B show the X-ray diffraction spectra for graphene and carboxylated graphene. In spectrum 2 A), a more intense peak at 2θ = 11.64° is observed, corresponding to the presence of graphene oxide, indicating an interplanar spacing of 7.8 × 10⁻^3^ nm. Additionally, a broader peak at 2θ = 24.35° suggests the reduction of graphene oxide to graphene [19,20,21,22,23]. In Figure 2B), a peak at 2θ = 11.80° corresponding to graphene oxide is noted, along with an additional peak at 2θ = 26.44°, which signifies an increase in crystallinity compared with the previous spectrum [20,22,23]. This enhancement in crystallinity may be attributable to the presence of carboxylic groups within the material.

### 3.3. Raman Spectroscopy

**Figure 3:**
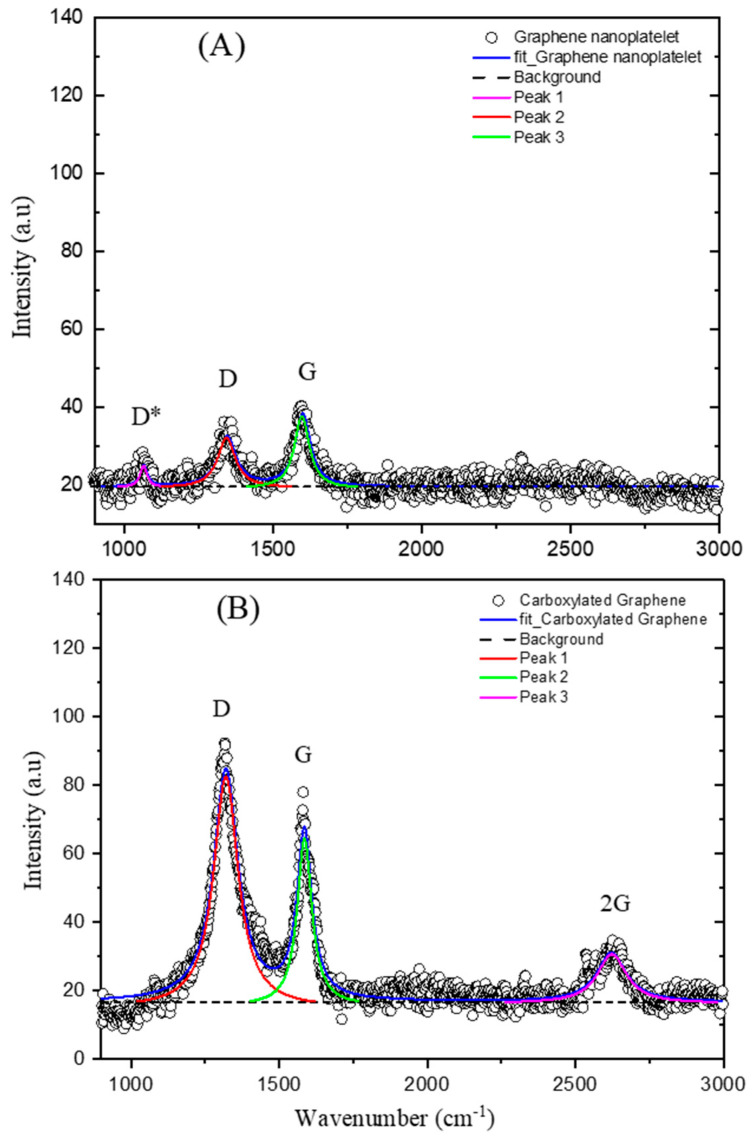
Raman spectra of (**A**) graphene nanoplatelets and (**B**) carboxylated graphene with the D*, D, G, and 2G band deconvolution.

The Raman spectra in Figure 3 display the characteristics of graphene nanoplatelets (A) and carboxylated graphene (B) obtained at room temperature. In Figure 3A, three distinct peaks are identified at 1065, 1345, and 1598 cm^−1^, corresponding to the material’s characteristic D*, D, and G modes [24]. In Figure 3B, the three typical bands associated with carbon-based materials can be seen, which correspond to the D band, G band, and 2D band, centered approximately at 1320, 1585, and 2640 cm^−1^, respectively [5]. The D* band indicates disordered graphitic lattices arising from sp^2^-sp^3^ bonding [24]. The G band signifies the planar structure of sp^2^-bonded carbon found in graphene, while the D band, often referred to as the defect or disorder band, results from a one-phonon lattice vibrational process. Additionally, the 2D band is linked to a two-phonon lattice vibrational process, resembling the behavior observed in other carbon-based materials, including nanotubes [5,24,25].

The intensity ratio of the D band to the G band (ID/IG) serves as a metric for assessing the disorder within graphene. Specifically, this ratio is 0.7 for graphene nanoplatelets and 1.3 for carboxylated graphene, as summarized in Table 1. The ID’/ID ratio is also utilized to investigate the types of defects present. This ratio is 1.3 for graphene, which indicates defects in the bonds, whereas for carboxylated graphene, it suggests the presence of vacancies within the hexagonal structure [17]. This finding aligns with the XRD pattern, demonstrating that the carboxylation process contributes to an increase in amorphism.

### 3.4. SEM and EDS

**Figure 4:**
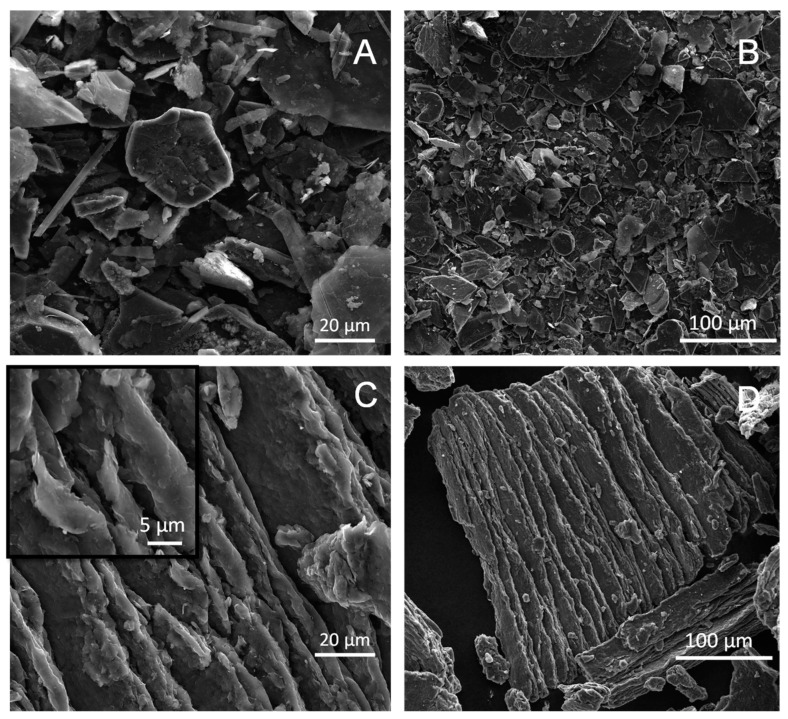
SEM images of (**A**,**B**) graphene and (**C**,**D**) carboxylated graphene.

The SEM images in Figure 4 illustrate the characteristics of graphene and carboxylated graphene. The micrographs (A and B) show that the graphene powder exhibits a flake structure with non-uniform sizes, featuring a slightly rough surface and numerous noticeable folds. In contrast, carboxylated graphene (CG), shown in Figure 4C,D, demonstrates improved uniformity compared with graphene. The overall structure of the CG underwent significant alteration, with the thin plates displaying a well-exfoliated, wrinkled, and crushed morphology. Furthermore, the surface of the synthesized CG appears smoother, more transparent, and thinner than conventional graphene. These findings confirm the successful synthesis of CG via reaction in an acidic medium [17,26].

**Figure 5:**
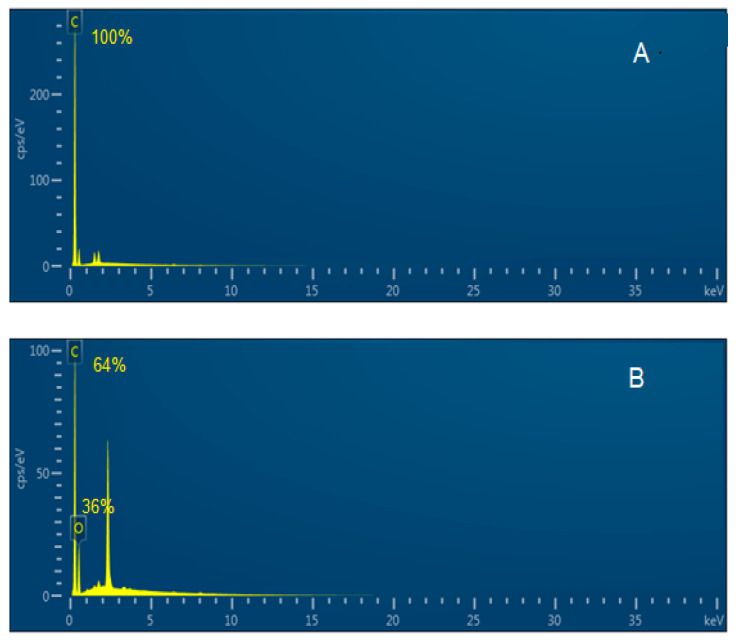
EDS spectrum of (**A**) graphene and (**B**) carboxylated graphene.

Energy-dispersive spectroscopy combined with scanning electron microscopy (SEM-EDS) was used to confirm the success of the synthesis, as shown in Figure 5. The graphene spectrum reveals the presence of only carbon, indicating the high purity of this sample. In contrast, the elemental composition of carboxylated graphene (CG) is characterized by distinct peaks for carbon and oxygen, suggesting the presence of carboxylic groups that are available for subsequent binding.

### 3.5. Electrochemical Properties

**Figure 6:**
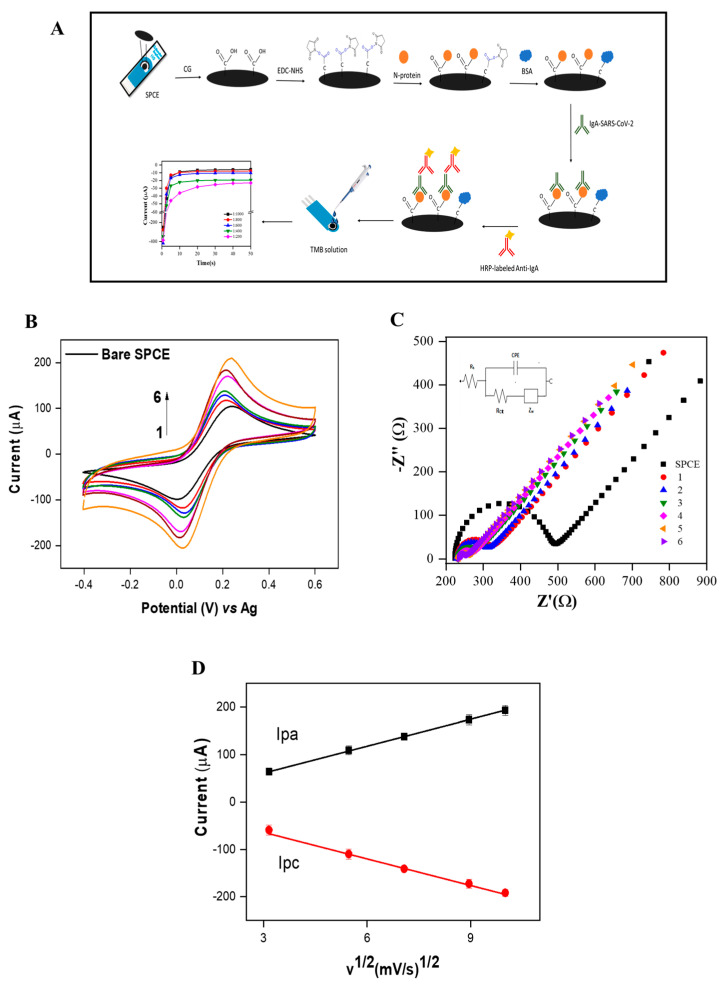
(**A**) Schematic representation of biosensor fabrication steps and IgA-SARS-CoV-2 determination, (**B**,**C**) CV and EIS for deposition of carboxylated graphene layers in 5 mmol L^−1^ [Fe(CN)6]^3−/4−^, prepared in 0.1 mol L^−1^ KCl. (**D**) The linear relationship between the current and square root of sweep speed (10–100 mV s^−1^) for modified SPCE with 3 depositions of CG.

Chemically modified electrodes that incorporate functional groups offer significant advantages over bare electrodes. Enhancing commercially available electrodes to achieve better physical distribution, increased electrochemical signals, and an improved immobilized surface is possible. Six depositions were tested to determine the optimal number for enhancing electrochemical signals. Each deposition involved applying 5 µL of a 2 mg/mL carboxylated graphene (CG) solution onto the surface of the screen-printed carbon electrode (SPCE). The fabrication steps of the electrochemical biosensor using CG are represented in Figure 6A. The electrochemical behavior of the CG-modified electrodes was assessed using the cyclic voltammetry (CV) technique, as shown in Figure 6B.

The electrochemical signal notably increases with each additional layer of carboxylated graphene (CG) applied, indicating that its presence significantly enhances signal intensity because of the material’s high electrical conductivity and surface area [27,28]. For the bare screen-printed carbon electrode (SPCE), the redox peaks displayed a separation of 0.236 V. Following the application of CG modification layers, the peak currents for the CG/SPCE improved by 12% to 102%. Additionally, the anodic and cathodic peak potentials converged, leading to a decrease in peak-to-peak separation (ΔEp), which varied from 0.16 to 0.2 V. This change suggests enhanced electron transfer between the electrochemical probe and the modified electrode surface, thereby improving the electrochemical performance of the SPCE. With CG modification, the capacitive current increased because of the accumulation of electrons on the electrode surface, enhancing the charge of the electrical double layer.

The electrochemical impedance spectroscopy (EIS) analysis (Figure 6C) revealed changes in charge transfer resistance, as depicted in the Nyquist plot. These diagrams can be fitted with an equivalent circuit model (inset Figure 6C), where RS represents the resistance of the electrolyte solution, CPE indicates the constant phase element, Rct is the electron-transfer resistance, and ZW reflects the Warburg element related to the diffusion behavior of the redox couple ([Fe(CN)_6_]^4^⁻/[Fe(CN)_6_]^3^⁻) at the interface from the bulk of the electrolyte. The semicircle observed at high to intermediate frequencies corresponds to the kinetic charge transfer process. In contrast, the straight line at low frequencies represents the diffusion barrier concerning the mass transfer of the redox couple [29]. The EIS spectra displayed clear distinctions in the signal after integrating each layer onto the SPCE surface. Starting with the bare SPCE, which exhibited an Rct of 240 Ω, a small diameter at high frequencies indicated some resistance to electron transfer. After modification with CG, the resistance gradually decreased (Rct = 19 Ω after six CG depositions), as confirmed by the small semicircle and straight line, indicating a diffusion process. This improved conductivity enhanced the electron transfer between the solution and the modified electrode surface. Three GC layers were selected as suitable for the biosensor assembly based on the results obtained.

Experiments utilizing various scan rates assessed the reversibility and transport nature associated with the CG-modified SPCE (Figure 6D). The cyclic voltammetry (CV) analysis characterized the SPCE modified with three layers of CG, demonstrating strong electrochemical performance. The peak separation (∆Ep) remained nearly constant as the scan rate increased, suggesting that the reaction approached a reversible system. As shown in the results, the anodic peak currents increased linearly with the square root of the scan rate, yielding an R^2^ value of 0.99 for both oxidation and reduction processes. Compared with photoreduction or multi-step chemical oxidation methods, which involve longer processing times, harsher conditions, and additional reagents, the applied CG synthesis method is more straightforward and reproducible. Electrochemical analyses and consistent functionalization observed via SEM, EDS, and Raman spectroscopy demonstrate its practical applicability and suitability for large-scale production.

### 3.6. Electrochemical Immunosensor Based in CG

The biosensor developed in this study builds upon the foundational work of Freire et al. [16]; however, the assembly steps were re-evaluated through electrochemical techniques, specifically cyclic voltammetry (CV) and electrochemical impedance spectroscopy (EIS). The experimental variables were also optimized to enhance the device’s performance, ensuring improved sensitivity and reliability in detecting target analytes.

### 3.7. Detection of IgA-SARS-CoV-2

**Figure 7:**
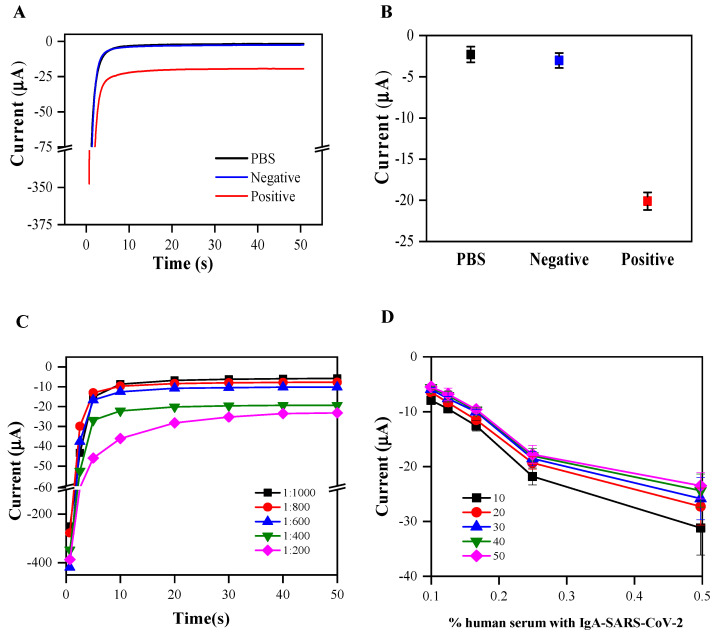
(**A**,**B**) Chronoamperograms were recorded with PBS, as well as negative and positive controls. (**C**) Chronoamperometric response was measured for different concentrations of human serum with IgA-SARS-CoV-2. (**D**) Current values registered from 10 to 50 s for different human serum concentrations with IgA-SARS-CoV-2.

Graphene is a widely utilized carbon nanomaterial renowned for its exceptional electrical conductivity and large surface area, surpassing carbon nanotubes and graphite. While graphene can significantly enhance the electrical conductivity of composite materials, it also leads to elevated capacitive currents from screen-printed electrodes (SPEs), often exceeding 1 µA. A fixed potential must be established during electrochemical measurements to reduce the contribution of capacitive currents. Consequently, chronoamperometry has been proposed as the analytical detection method for biosensors utilizing SPE modified with this material. In detecting IgA-SARS-CoV-2 [16,30], human serum samples from negative and positive COVID-19 patients were used. Various concentrations of positive serum were tested, adjusting the dilution factor from 1:1000 to 1:200 *v*/*v* in 1X PBS. As illustrated in Figure 7A, chronoamperometric measurements were conducted at −0.19 V (reduction potential) versus a pseudo-Ag reference for 50 s. A rapid decrease in current values was observed during the initial 10 s, attributable to electrode polarization. A subsequent stabilization phase occurred, during which the current values approached a plateau. The chronoamperograms for the blank (PBS) and the negative sample showed similar current values close to zero, indicating the absence of an enzymatic reaction. In contrast, the positive sample exhibited a notable increase in reduction currents, as depicted in Figure 7B.

**Figure 8:**
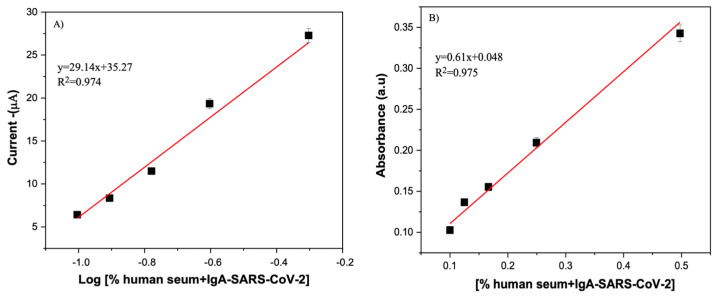
Calibration curve for IgA-SARS-CoV-2 determination through (**A**) CA technique and (**B**) ELISA technique. Standard error bars correspond to measurements made on three replicates of each concentration (*n* = 3).

The response of the positive sample at different concentrations is illustrated in Figure 7C, which demonstrates a linear relationship between the percentage of human serum containing IgA-SARS-CoV-2 and the current values. The current was analyzed over a time frame from 10 to 50 s, as shown in Figure 7D, revealing consistent linear behavior across all intervals; however, this linearity decreased at the highest concentration of IgA-SARS-CoV-2, potentially because of electrode passivation associated with TMB precipitation [16,31,32]. The calibration curve was plotted using the absolute values of stable current recorded at 20 s against the logarithm of % human serum containing IgG-SARS-CoV-2. The performance was evaluated at 1:1000, 1:800, 1:600, 1:400, and 1:200 *v*/*v* dilution factors. The regression equation obtained was y= 29.14x + 35.27, with an R^2^= 0.974, as depicted in Figure 8A.

The limit of detection (LOD) was calculated following the recommendations of the International Union of Pure and Applied Chemistry, using the formula LOD = 3.3 × SD/m (where SD is the standard deviation of the blank and m is the slope of the calibration curve) [33]. This limit was determined to be 4.59 µA, corresponding to 0.0624% of IgG-SARS-CoV-2, which equates to a dilution factor of 1:1601 *v*/*v*. Experiments were performed using the ELISA technique to validate the method proposed in this study. The calibration curve obtained (Figure 8B) plotted absorbance against % human serum containing IgG-SARS-CoV-2. The results showed a linear relationship within the same concentration range as our method (from 1:1000 to 1:200), yielding a LOD of 0.0771% of IgG-SARS-CoV-2, equivalent to a dilution factor of 1:1296 *v*/*v*. The curve equation was y = 0.61x + 0.048, with an R^2^ = 0.975. The immunosensor and Elisa exhibited similar R^2^ values, though the LOD of the immunosensor was 1.2 times higher. Additionally, the immunosensor method offered notable advantages, requiring only half the application time, and using smaller amounts of immobilized protein and sample volume compared to the ELISA assay.

To evaluate the biosensor stability, the BSA/N-protein/EDC-NHS/CG/SPCE was stored at 4 °C for seven days, and measured the electrochemical response by cyclic voltammetry in 5 mmol L^−1^ [Fe(CN)6]^3−/4−^ + 0.1 mol L^−1^ KCl (Figure S1). The profile before and after did not evidence significant changes, with approximately 95% of the values of Ipa, Ipc, and ∆E being conserved, confirming to be stable at this time.

## 4. Conclusions

This study demonstrates a successful and efficient synthesis of CG, producing a conductive nanomaterial optimized for electrochemical biosensors. The applied synthesis method for CG is highly cost-effective. Using readily available reagents such as nitric and sulfuric acid, combined with straightforward processing steps, minimizes material and operational costs. Dissimilar to other techniques requiring expensive catalysts, specialized equipment, or extensive purification steps, this approach ensures accessibility for research laboratories and industrial-scale production. The affordability of this method further supports its potential for widespread adoption in biosensor applications.

The CG-modified screen-printed SPCEs demonstrated excellent stability and high sensitivity in detecting SARS-CoV-2 IgA antibodies. Electrochemical analysis revealed a detection range of 1:1000 to 1:200 *v*/*v*, with a detection limit of 1:1601 *v*/*v* in PBS buffer, underscoring the biosensor’s potential for sensitive diagnostic applications. The CG/SPCEs exhibited a strong correlation between antibody concentration and current response (R^2^ = 0.974), attributed to the improved electron transfer facilitated by the high surface area and conductivity of CG. Furthermore, EIS analysis indicated a significant reduction in charge transfer resistance, confirming improved electron flow and an enhanced signal response due to CG modification.

Compared with conventional ELISA methods, the CG-based biosensor exhibited similar accuracy while providing significant operational benefits, such as faster response times, reduced reagent usage, and smaller sample volume requirements. These advantages make it particularly well-suited for point-of-care diagnostics, especially in resource-limited settings. Additionally, the chronoamperometric analysis demonstrated a stabilized current response within just 10 s, further highlighting the platform’s efficiency and suitability for rapid testing applications.

Considering the continuing need for efficient COVID-19 diagnostic tools, the CG/SPCE-based biosensor presents a promising and versatile solution with the potential to be adapted for detecting a broader spectrum of respiratory pathogens. Future research should explore the application of this platform for identifying additional viral biomarkers, further enhancing its utility in clinical diagnostics and epidemiological surveillance. This study not only emphasizes the significant potential of CG in biosensing applications but also highlights its suitability for scalable, cost-effective, and accurate diagnostics, particularly in resource-constrained settings.

## Figures and Tables

**Table 1:** Details of Raman spectra of nanoplatelets and carboxylated graphene. The peak intensity ratio of ID/IG.

Samples	D Peak (cm^−1^)	β (cm^−1^)	G Peak (cm^−1^)	β (cm^−1^)	I_D_/I_G_
Graphene nanoplatelets	1345	12.8	1598	18.5	0.7
Carboxylated graphene	1322	67.6	1586	49.0	1.3

## Data Availability

The data used to support the findings of this study are available from the corresponding author upon request.

## Supplementary Materials

The following supporting information can be downloaded at https://www.mdpi.com/article/10.3390/bios15010034/s1, Figure S1. Cyclic voltammograms in 5 mmol L^−1^ [Fe(CN)_6_]^3−/4−^ + 0.1 mol L^−1^ KCl of the biosensor before and after storage at 4 °C for seven days.
