# Supplementary material for: Carboxylated Graphene: An Innovative Approach to Enhanced IgA-SARS-CoV-2 Electrochemical Biosensing
Source: Biosensors (Basel). 2025 Jan 9;15(1):34. doi: 10.3390/bios15010034 (PMC11764328; doi:10.3390/bios15010034)
Supplement: Supplementary file 1 [file biosensors-15-00034-s001.zip › biosensors-3351970-supplementary.pdf]

Carboxylated Graphene: An Innovative Approach to Enhanced IgA-SARS-CoV-2 Electrochemical Biosensing

Luciana de Souza Freire [1,†], Ariamna María Dip Gandarilla [1,†], Yonny Romaguera Barcelay [1], Camila Macena Ruzo [1], Barbara Batista Salgado [2], Ana P. M. Tavares [3], Francisco Xavier Nobre [4], Julio Nino de Souza Neto [5], Spartaco Astolfi-Filho [5], Ștefan Țălu [6,*], Pritesh Lalwani [2], Niranjan Patra [7] and Walter Ricardo Brito [1,5,*]

[1] LABEL–Laboratório de Bioeletrônica e Eletroanalítica, Central Analítica Multidisciplinar, Universidade Federal do Amazonas, Manaus 69067-005, Amazonas, Brazil; lsfreire@ufam.edu.br (L.d.S.F.); ariamna@ufam.edu.br (A.M.D.G.); yonny.barcelay@uc.pt (Y.R.B.); camilarz@ufam.edu.br (C.M.R.)

[2] Instituto Leônidas e Maria Deane (ILMD), Fiocruz Amazônia, Manaus 69029-520, Amazonas, Brazil; barbara.salgado@fiocruz.br (B.B.S.); pritesh.lalwani@fiocruz.br (P.L.)

[3] CENTI, Centro de Nanotecnologia e Materiais Técnicos, Funcionais e Inteligentes, R. Fernando Mesquita 2785, 4760-034 Vila Nova de Famalicão, Portugal; ana.p.tavares90@gmail.com

[4] Group of Energy Resources and Nanomaterials (GREEN Group), Department of Chemistry, Environment, and Food (DQA), Federal Institute of Education, Science and Technology of Amazonas, Campus Manaus Centro, Manaus 69020-120, Amazonas, Brazil; francisco.nobre@ifam.edu.br

[5] PPGBIOTEC—Programa de Pós-Graduação em Biotecnologia, Universidade Federal do Amazonas, Manaus 69067-005, Amazonas, Brazil; ninobio@ufam.edu.br (J.N.d.S.N.); spartaco.biotec@gmail.com (S.A.-F.)

[6] The Directorate of Research, Development, and Innovation Management (DMCDI), The Technical University of Cluj-Napoca, 400020 Cluj-Napoca, Romania

[7] Department of Chemistry, Koneru Lakshmaiah Education Foundation, Greenfield, Vaddeswaram 522502, Andhra Pradesh, India; patraji@gmail.com

[*] Correspondence: stefan.talu@auto.utcluj.ro (Ș.Ț.); wrbrito@ufam.edu.br (W.R.B.); Tel.: +55-92981379920 (W.R.B.)

[†] These authors contributed equally to this work.

Supplementary Materials

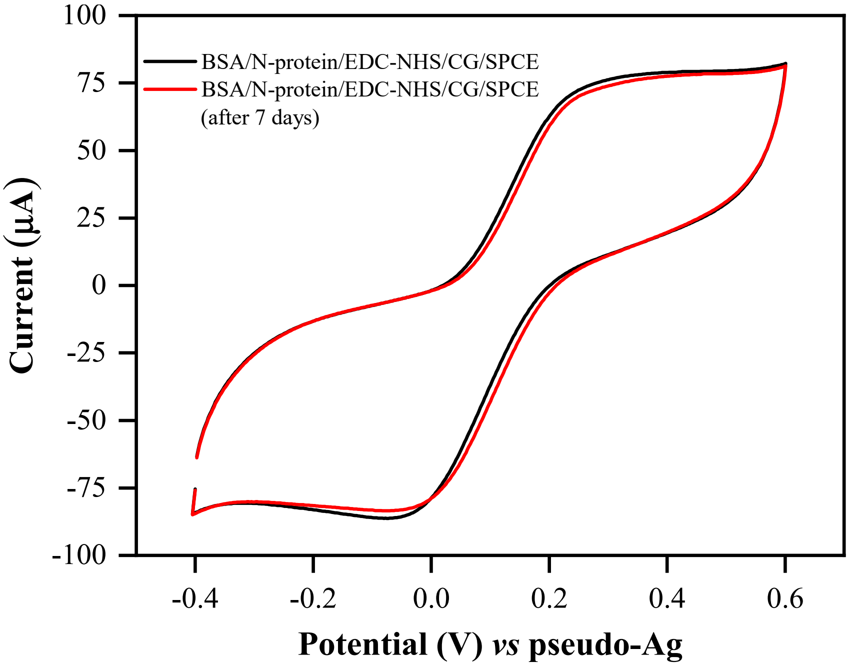

Figure S1. Cyclic voltammograms in 5 mmol L^{-1} $[Fe(CN)_6]^{3-/4-}$ + 0.1 mol L^{-1} KCl of the biosensor before and after storage at 4 °C for seven days.
